# Regulation of the Aurora-A gene following topoisomerase I inhibition: implication of the Myc transcription Factor

**DOI:** 10.1186/1476-4598-9-205

**Published:** 2010-08-03

**Authors:** Sandy Courapied, Julia Cherier, Arnaud Vigneron, Marie-Bérangère Troadec, Sandrine Giraud, Isabelle Valo, Claude Prigent, Erick Gamelin, Olivier Coqueret, Benjamin Barré

**Affiliations:** 1 Cancer Center Paul Papin; INSERM U892; Angers, France; 2 Cancer Center Paul Papin; Biopathology Department, France; 3 CNRS UMR 6061, Université de Rennes I, IFR140, France; 4 EA 3142, Université d'Angers, France

## Abstract

During the G2 phase of the cell cycle, the Aurora-A kinase plays an important role in centrosome maturation and progression to mitosis. In this study, we show in colorectal cell lines that Aurora-A expression is downregulated in response to topoisomerase I inhibition. Using chromatin immunoprecipitation assays, we have observed that the Myc transcription factor and its Max binding partner are associated with the Aurora-A promoter during the G2 phase of the cell cycle. RNA interference experiments indicated that Myc is involved in the regulation of the Aurora-A gene. Following topoisomerase I inhibition, the expression of Myc decreased whereas Mad was upregulated, and the association of Myc and Max with the promoter of the kinase was inhibited. In parallel, an increased association of Mad and Miz-1 was detected on DNA, associated with an inhibition of the recruitment of transcriptional coactivators. Interestingly, a gain of H3K9 trimethylation and HP1γ recruitment was observed on the Aurora-A promoter following sn38 treatment, suggesting that this promoter is located within SAHF foci following genotoxic treatment. Since Aurora-A is involved in centrosome maturation, we observed as expected that topoisomerase I inhibition prevented centrosome separation but did not affect their duplication. As a consequence, this led to G2 arrest and senescence induction.

These results suggest a model by which the Aurora-A gene is inactivated by the G2 checkpoint following topoisomerase I inhibition. We therefore propose the hypothesis that the coordinated overexpression of Myc and Aurora-A, together with a downregulation of Mad and Miz-1 should be tested as a prognosis signature of poor responses to topoisomerase I inhibitors.

## Background

The response to genotoxic treatments relies to a large extent on the activation of the ATM and ATR kinases and on the consequent upregulation of chk1 and chk2 signaling [1-3]. Among numerous substrates, this signaling network leads to the activation and stabilization of the p53 pathway which induces apoptosis or cell cycle arrest [4]. In addition to this protective pathway, others checkpoints are also involved in the control of the progression towards mitosis. At the G1/S transition, chk1/2 activation promotes the degradation of cdc25A by the SCF^βTCRP ^complex, leading to cdk2 inactivation and G1 phase arrest [5]. During G2 and mitosis, the inhibition of cdc25C by chk1/2 induces the inactivation of cyclin B-cdk1 complexes [6,7], whereas the BubR1, Mad1 or Mad2 proteins can prevent anaphase following spindle checkpoint activation [8].

In association with the cyclin B-cdk1 complexes and cdc25C, the Aurora-A serine/threonine kinase is also essential for progression to mitosis [9,10]. This protein localizes in early G2 to duplicated centrosomes where it plays an important role in their maturation, separation and in the consequent assembly of the spindle apparatus. Illustrating its essential role in spindle organization, the inactivation of Aurora-A leads to the generation of spindle defects, mitotic catastrophe and aneuploidy [10,11]. Importantly, a high expression of the kinase, often due to gene amplification at 20q13, has been detected in several epithelial tumors such as breast, ovarian, gastric, pancreatic and colorectal cancers [9]. In addition, the overexpression of Aurora-A transforms NIH3T3 fibroblasts, probably as a consequence of abnormal mitosis and inactivation of the p53 tumor suppressor gene [12]. An abnormal expression of this kinase is therefore believed to play an important role in cell transformation and genetic instability.

Despite recent studies [13], the regulation of Aurora-A during DNA damage remains most of the time to be characterized. In this study, we show that topoisomerase I inhibitors, one the main drug used in the treatment of colorectal cancers [14,15], induced a downregulation of Aurora-A expression and prevented centrosome separation. In normal conditions, we found that the Myc transcription factor binds to the promoter of this gene in association with Max. Following topoisomerase I inhibition, Myc/Max binding is inhibited, Mad and Miz-1 associate with this promoter and this is associated with transcriptional downregulation.

Altogether, these results indicate that Aurora-A is downregulated in response to topoisomerase I inhibition. We propose that this inhibition plays an important role during the G2 checkpoint in parallel to p53 induction and cdc25C inactivation.

## Methods

### Reagents

Polyclonal anti-phospho p53 (SC-11764-R), anti-c-myc (SC-764), anti-p21waf1 (SC-397), monoclonal anti-p53 (SC-98), anti-max (C17) (SC-197), anti-mad1 (C19) (SC-222), anti-CBP (A22) (SC369), anti-RNA polymerase II (N20) (SC899), anti-HP1 (S-19) and anti-miz1 (H190) (SC-22837) were obtained from Santa Cruz Biotechnology (Santa Cruz). Monoclonal anti-α and γ-tubulin were obtained from Sigma, anti-H3K9me3 (07-442) and anti-H3-Ac (06-599) were from Upstate. All statistical analysis have been performed with the Graphpad software.

### Primers

Total RNA was isolated from cell lines with TRIzol reagent (Invitrogen) and expression was measured by real time PCR analysis using GADPH or RPLPO as a normalization standards. The following primers were used:

Aurora A: For 5'-GATCAGCTGGAGAGCTTAAA-3', Rev 5'-GAGGCTTCCCAACTAAAAAT-3'; c-Myc: For 5'-ATTCTCTGCTCTCCTCGAC-3', Rev 5'-GTAGTTGTGCTGATGTGTGG-3'; Max: For 5'-ACGAAAACGTGGGACCACATC-3', Rev 5'-GTGTGTGGTTTTTCCCGCATAT-3'; Mad: For 5'-GGTTCGGATGAACATCCAG-3', Rev 5'-GGCATCTCTGTCCTTGTTATTGT-3'; Miz-1: For 5'-GGCAAACTGTCAGAAAAGAGTAGC-3', Rev 5'-CGCTGCTGGTTCAGCTGTT-3'; p21WAF1: For 5'-GCTCCTTCCCATCGCTGTCA-3' Rev 5'-TCACCCTGCCCAACCTTAGA-3'; GAPDH: For 5'-GAAGGTGAAGGTCGGAGTC-3', Rev 5'-GAAGATGGTGATGGGATTTC-3; 3' RPLPO: For 5'-AACCCAGCTCTGGAGAAACT-3' and Rev 5'-CCCCTGGAGATTTTAGTGGT-3'

### Cell lines and treatment

The human colorectal cell lines HT29 (HTB-38) and HCT116 (CCL-247) (ATCC, Manassas, VA20108, USA) were cultured in RPMI 1640 medium (Lonza Walkersville, USA). Cell lines were supplemented with 10% fetal bovine serum (PAA laboratories GmbH, Austria). Cells grown in 3% FBS medium were immediately treated with sn38 (5 ng/ml, 12.5 nM) for 48 h. Note that this treatment should be done before complete cell adhesion so that every cell can incorporate the drug before entering the next S phase. To choose this concentration, clonogenic assays were performed to determine the concentration that kill all cells after 10 days. For HCT116 cells, 5 ng/ml induced 100% mortality.

### Chromatin Immunoprecipitation Assay (ChIP)

Cells, grown to 60% confluence, were treated or not as indicated and then washed and cross-linked with 1% formaldehyde at room temperature for 8 min essentially as previously described [16,17]. Reaction was stopped with 10 ml of 125 mM glycin solution. Cells were washed with cold PBS and lysed in 500 μl of lysis buffer (1% SDS, 10 mM EDTA, 50 mM Tris-HCl pH 8.1, 1 mM PMSF, 5 mM NaF, 5 mM Na3VO4, 2 μg/ml leupeptin, 5 μg/ml aprotinin, 1 μg/ml pestatin), and sonicated five times for 20 secondes each. Supernatants were then recovered by centrifugation at 12 000 rpm for 10 min at 4°C, diluted once in dilution buffer (1% Triton X-100, 2 mM EDTA, 150 mM NaCl, 20 mM Tris-HCl pH 8.1) and subjected to one round of immunoclearing for 2 h at 4°C with 2 μg of sheared salmon-sperm DNA, and 20 μl of proteinG-agarose coated with salmon sperm DNA (Millipore) (of 50% slurry). Immunoprecipitation was performed overnight with specific antibodies and IgG control, and then 2 μg of sheared salmon-sperm DNA and 20 μl of proteinG-agarose coated with salmon sperm DNA (Millipore) (of 50% slurry) were further added for 1 h at 4°C. Note that immunoprecipitations were performed in the presence of 0,1% Igepal CA-630. Immunoprecipitates were washed sequentially for 10 min each in TSE I (0.1% SDS, 1% Triton X-100, 2 mM EDTA, 20 mM Tris-HCl pH 8.1, 150 mM NaCl), TSE II (0.1% SDS, 1% Triton X-100, 2 mM EDTA, 20 mM Tris-HCl pH 8.1, 500 mM NaCl), and Buffer 3 (250 mM LiCl, 1% NP-40, 1% deoxycholate, 1 mM EDTA, 10 mM Tris-HCl pH 8.1). Beads precipitates were then washed once with TE buffer and eluted once with 1% SDS, 100 mM NaHCO3. For Re-ChIP experiments 25 μl of ReChIP buffer (Dilution Buffer, 10 mM DTT) was added to beads following washes and incubated at 37°C for 30 minutes. The sample was then diluted 40 times in dilution buffer and immunoprecipitations, washes and elution were performed as before ([18]). Eluates were heated at 65°C for 6 hours to reverse the formaldehyde cross-linking. DNA was precipitated using classical procedures. Real-time PCR was used for ChIP analysis and quantification. The ChIP have been calculated as binding to region of interest/IgG control, divided by binding to negative control region/IgG control. The following primers were used:

region -668/-400 of the Aurora A promoter: For 5'-GAT GCCCCCTCACTATATGC-3', Rev 5'-AGGAGAGAGCGGGATACCAA-3'; region -114/+161 of the Aurora A promoter: For 5'-AGGTCTGGCTGGCCGTTG-3', Rev 5'-CCTCGTCCGCCACTGAGATAT-3' Control region -1701/-1399 of the Aurora A promoter For 5'-ACTCCAGATCCCTCAGCTTAACCA-3' Rev 5'-CAAGTTATGGGACGGTGAACG-3'

### Other assays

Transient transfections, siRNA knockdown, RNA extraction, semi-quantitative and quantitative reverse transcription-polymerase chain reaction, protein extracts and western blots were all performed as described previously [17,19]. All experiments were performed a minimum of three times before calculating means and standard deviations as shown in figures.

## Results

### Topoisomerase I inhibition induced a downregulation of Aurora-A expression

**Figure 1:**
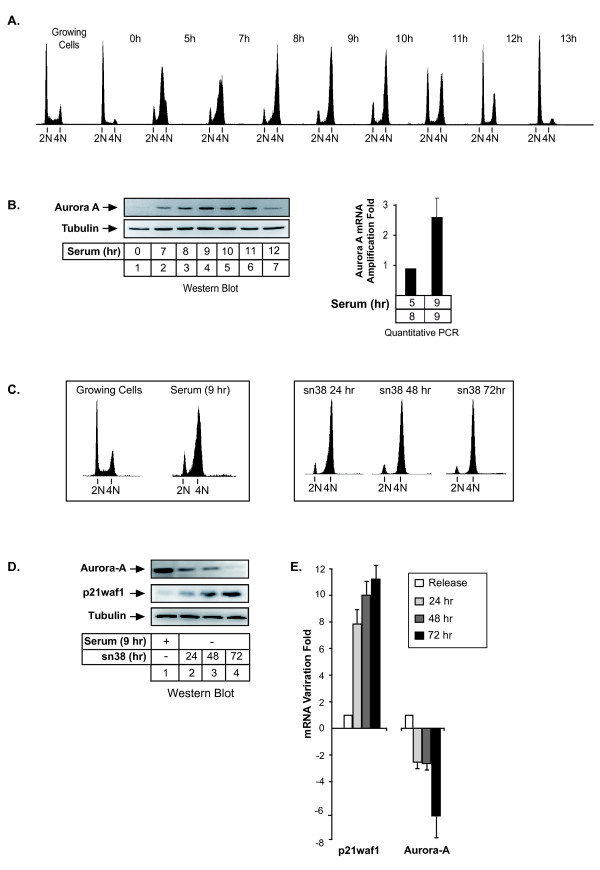
**Topoisomerase I inhibition induced a downregulation of Aurora-A expression**. A. HCT116 were synchronized in G1/S with hydroxyurea and released for the indicated times in growth medium complemented with 3% serum. DNA content was analyzed by flow cytometry analysis. B. Aurora-A expression was analyzed in these conditions by western blot (lanes 1-7), or quantitative RT-PCR (B, lanes 8-9) (n = 3 +/- sd). C. HCT116 cells were synchronized in the G2 phase of the cell cycle following treatment with hydroxyurea and serum stimulation for 9hr in growth medium (serum 9 hr) or treated with sn38 (5 ng/ml, 12.5 nM). DNA content was then analyzed by flow cytometry and propidium iodide staining. D-E. Aurora-A expression was measured by western blot analysis (D, lanes 1-4) or quantitative RT-PCR (E) following treatment or cell synchronisation. p21waf1 and tubulin expressions were used as controls (n = 3).

We first wanted to confirm in colorectal cell lines that Aurora-A was mainly expressed during the G2 phase of the cell cycle. To this end, HCT116 cells were synchronized in G1/S with hydroxyurea, washed and then grown again in serum for 5 to 13 hr. Under these conditions, FACS analysis showed that cells were synchronized after 8-9 hr in the G2 phase of the cell cycle and that they enter the next G1 phase after 12-13 hr of serum release (Figure 1A). As expected, we observed that Aurora-A was expressed in G2, both at the protein (Figure 1B, lanes 1-7) and mRNA levels (Figure 1B, lanes 8-9). The same results were obtained in a second colorectal cell line, the HT29 cells and with different kinds of synchronization such as double thymidine block and serum starvation (data not shown).

**Figure 2:**
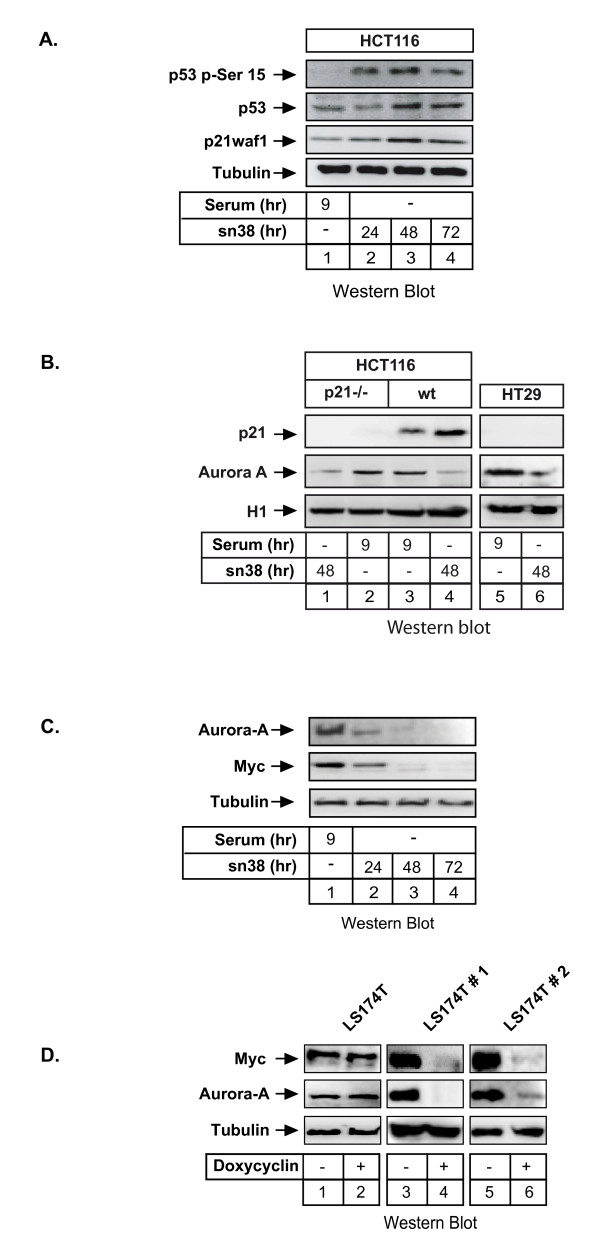
**Myc regulates the Aurora-A promoter**. A. HCT116 cells were synchronized as above, total cell extracts were prepared and analyzed using antibodies directed against p21waf1, p53 or its serine 15 phosphorylated form (n = 3). B. HCT116 p21-/- or HT29 cells (presenting a mutated form of p53) were treated as described above and Aurora-A and p21waf1 expressions were measured by western blot using tubulin as a control (n = 3). C. HCT116 cells were synchronized in the G2 phase of the cell cycle (serum 9hr) or treated with sn38 for the indicated times. The expression of Myc and Aurora-A was analyzed by western blot on total cell extracts (n = 3). D. LS174T cells were grown in the absence or presence of doxycyclin as indicated. Myc and Aurora expressions were then analyzed by western blot analysis (n = 3). Two different clones of the LS174T colorectal cell line (named LS174T#1 and LS174T#2) were used, each expressing a doxycyclin-inducible expression vector that drives the expression of different siRNAs.

To determine whether topoisomerase I inhibition has any influence on Aurora-A expression, HCT116 cells were treated with sn38, the active metabolite of irinotecan [15]. Under these conditions, control cells were synchronized in the G2 phase of the cell cycle after 48-72 hr (Figure 1C, note that serum 9hr means G1/S synchronization followed by serum stimulation for 9 hr). Although this was the cell cycle stage when Aurora-A expression was supposed to be maximal, results indicated that the expression of the kinase was downregulated in response to sn38, both at the protein (Figure 1D, compare lane 1 with lanes 2-4) and mRNA levels (Figure 1E, normal mRNAs expression in G2 was normalized to 1). As a control, the p21waf1 mRNA increased as expected following genotoxic treatment. Finally, these experiments have also been repeated in a different colorectal cell line and sn38 also downregulated Aurora-A in HT29 cells (data not shown and see below Figure 2B).

Altogether, these results indicate that topoisomerase I inhibitors such as sn38 induced a downregulation of Aurora-A expression.

### Myc binds to the promoter of the Aurora gene and is involved in its regulation

Following sn38 treatment, we observed as expected in HCT116 cells that p53 was stabilized and phosphorylated on its serine 15 residue. Consequently, p21waf1 level was also enhanced in response to drug treatment (Figure 2A, lanes 1-4). To check whether Aurora-A downregulation was dependent on the p53-p21 pathway [20,21], we used the HCT116 p21-/- derivative cell line in which both p21waf1 alleles have been deleted by homologous recombination [22]. Results showed that sn38 reduced Aurora-A expression in HCT116 p21-/- cells (Figure 2B, lanes 1-4). The same effect was observed in the HT29 cell line that contains a mutated form of p53 (Figure 2B, lanes 5-6). These results indicate that Aurora-A downregulation is not cell-type specific and is independent of the p53-p21 pathway.

During the course of this study, we noticed that the expression of the c-Myc transcription factor was significantly reduced following topoisomerase I inhibition (Figure 2C, lanes 1-4). This suggested that c-Myc was involved in the regulation of the Aurora-A gene. To verify this hypothesis, we used doxycyclin-inducible expression vectors that stably drives the expression of two different Myc siRNAs in two different clones of the LS174T colorectal cell line. As previously shown [17], western blot analysis showed that doxycyclin induced a significant downregulation of c-Myc levels in the two clones (Figure 2D, lanes 4 and 6, top panel). Interestingly, we observed under these conditions that Aurora-A expression was inhibited upon c-Myc knockdown (Figure 2D, compare lanes 4 and 6 with lanes 3 and 5, middle panel). Note that c-Myc downregulation did not modify cell cycle distribution in the G2 phase of the cell cycle (data not shown) so that Aurora-A inhibition can not be explained by G0/G1 arrest.

**Figure 3:**
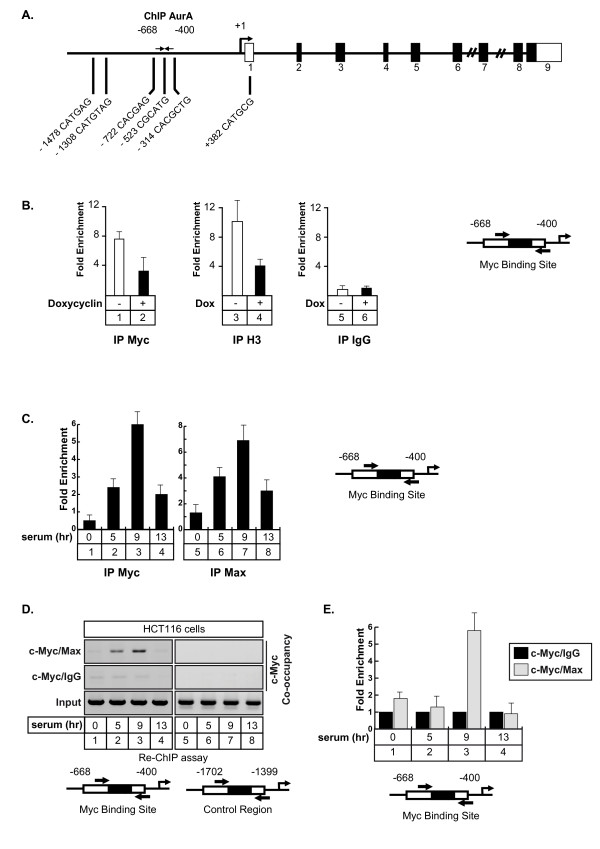
**Myc and Max are associated with the Aurora-A promoter**. A. Schematic representation of the potential Myc binding sites of the Aurora-A promoter. B. LS174T cells were treated or not with doxycyclin, soluble chromatin was immunoprecipitated with anti-Myc or anti-acetylated-H3 polyclonal antibodies and DNA samples were then amplified using primers that cover the -668/-400 region of the Aurora-A promoter. IgG immunoprecipitations were used as controls (n = 3 +/- sd). C. HCT116 were synchronized in G1/S with hydroxyurea and released for the indicated times in growth medium complemented with 3% serum. Soluble chromatin was immunoprecipitated with anti-Myc or anti-Max antibodies and DNA samples were then amplified using primers that cover the -668/-400 region of the Aurora-A promoter and quantified as compared to IgG immunoprecipitations (n = 3 +/- sd). D, E. The association of Myc and Max on the Aurora-A promoter was analyzed by a serial ChIP experiment. HCT116 were synchronized as described above, the soluble chromatin was immunoprecipitated with Myc antibodies, immune complexes were released and reimmunoprecipitated with IgG or Max antibodies. DNA samples were then amplified using primers that cover the -668/-400 region of the Aurora-A promoter and analyzed by semi-quantitative PCR (D) or quantitative PCR (E, n = 3 +/- sd).

Using the UCSC genome browser http://genome.ucsc.edu, we noticed that ChIP-ChIP experiments have already suggested that Myc can potentially bind to the Aurora-A promoter in Hela cells. Moreover, Ouyang and collaborators have shown by ChIP-seq that both c-Myc and N-Myc can be found associated with this gene in embryonic stem cells [23]. Effectively, transcription factor recognition site analysis of the Aurora-A promoter revealed the presence of non canonical E-boxes that could represent potential Myc binding sites (Figure 3A). To determine if Myc binds to the Aurora A promoter, its recruitment was analyzed by chromatin immunoprecipitation experiments (ChIP) in the LS174T cell line described above. Results presented Figure 3B, lanes 1-4, showed that Myc was effectively recruited to the -668/-400 region of the Aurora-A promoter and that this was associated with histone 3 acetylation (K9), which is indicative of gene transcription. Following siRNA induction and Myc downregulation, the binding of the transcription factor was downregulated and this inhibition was associated with histone H3 deacetylation (Figure 3B, lanes 2 and 4). As a control, no binding of a control IgG (Figure 3B, lanes 5-6), and Myc did not bind to the 5' part of the Aurora-A promoter (data not shown).

Myc is a basic helix-loop-helix zipper transcription factor that heterodimerizes with Max to activate gene transcription. Its activity is inhibited by Mad which associates with Max to recruit repressor complexes to promoters [24]. To determine if Myc and Max are associated with the Aurora-A promoter and if this association is cell cycle dependent, HCT116 cells were synchronized in G1/S with hydroxyurea, washed and then grown again in serum for 5 hr (S/early G2), 9 hr (G2) and 13 hr (next G1). ChIP experiments were then performed as described above. Results presented Figure 3C, lanes 3 and 7, indicate that the two proteins are effectively recruited to this promoter in the G2 phase of the cell cycle. To determine if the two proteins are associated on DNA, a serial ChIP experiment (Re-ChIP) was then performed. For this, the soluble chromatin was immunoprecipitated with Myc antibodies, the immune complexes were released with DTT and the chromatin was further divided into two aliquots and reimmunoprecipitated with IgG or Max antibodies. Under these conditions, subsequent Re-IPs with Max antibodies were able to immunoprecipitate the Aurora-A promoter whereas this was not the case with the control antibody (Figure 3D). Importantly, the association of the two proteins was only detected during the G2 phase of the cell cycle. ChIP result have been obtained by semi-quantitative PCR (Figure 3D, lanes 1-4) and quantified by quantitative-PCR (Figure 3E). As a control, the PCR analysis did not detect any occupancy of a control DNA region (Figure 3D, lanes 5-8) or of the proxymal promoter during the G1 phase of the cell cycle and at the G1/S transition (Figure 3D, lane 4 and 1).

We concluded from these results that the Myc/Max complex binds to the promoter of the Aurora A gene during the G2 phase of the cell cycle and that Myc is involved in the regulation of this gene.

### Topoisomerase I inhibition prevents the association of the Myc-Max complex with the Aurora-A promoter

**Figure 4:**
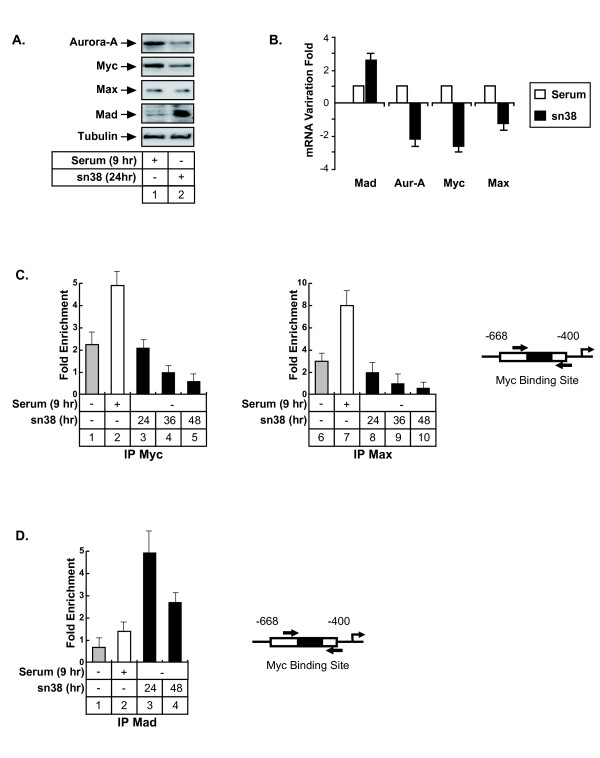
**Topoisomerase I inhibition prevents the association of Myc and Max with the Aurora-A promoter**. A. HCT116 were synchronized as described above and analyzed by western blot using antibodies directed against the indicated proteins (n = 3). B. Cells were treated as described above and the mRNAs expressions of Aurora-A, Myc, Max and Mad were evaluated by quantitative RT-PCR (n = 3 +/- sd). C-D. The association of Myc, Max and Mad with the Aurora-A promoter was analyzed in HCT116 by chromatin immunoprecipitation as described above (n = 3 +/- sd). ChIPs were performed using extracts isolated from growing cells (C, lanes 1 and 5, D lane 1), from cells synchronized in the G2 phase of the cell cycle (C, lanes 2 and 7, D lanes 2) or from cells treated by sn38 as indicated.

To determine the links between the Myc/Max/Mad pathway and the regulation of the Aurora-A gene following topoisomerase I inhibition, Max/Mad expression was first evaluated following sn38 treatment. Whereas no significant effect was observed on Max expression, Mad levels increased at the protein and mRNA levels (Figure 4A, lanes 1-2 and Figure 4B). As a control, Myc and Aurora-A expressions were downregulated as expected. To determine if the binding of these proteins to the Aurora-A gene was affected by sn38, their recruitment was analyzed by ChIP following treatment. Results showed that the recruitment of Myc and Max was inhibited following topoisomerase I inhibition (Figure 4C, compare lanes 2 and 5, 7 and 9). Note that a weak association of Myc and Max was detected in growing conditions, probably due to the percentage of cells, which are in the G2 phase of the cell cycle (Figure 4C, lanes 1 and 6). Interestingly, these proteins were also found associated with the initiation site, suggesting that the upstream and initiation regions might associate in a transcriptional loop (data not shown). Myc and Max bindings were also inhibited on this initiation site following sn38 treatment. To extend these observations, ChIP experiments were then performed to analyze the recruitment of the Mad protein. In growing conditions or during the G2 phase of the cell cycle, Mad was not found associated with the Aurora-A promoter. Interestingly, when cells were treated with sn38, this protein was significantly recruited to this gene (Figure 4D, lanes 3-4).

Altogether, we concluded from these results that the Myc/Max complex binds to the promoter of the Aurora-A gene in the G2 phase of the cell cycle and that this binding is inhibited upon topoisomerase I inhibition.

### Topoisomerase I inhibition promotes Miz-1 recruitment to the Aurora-A promoter

**Figure 5:**
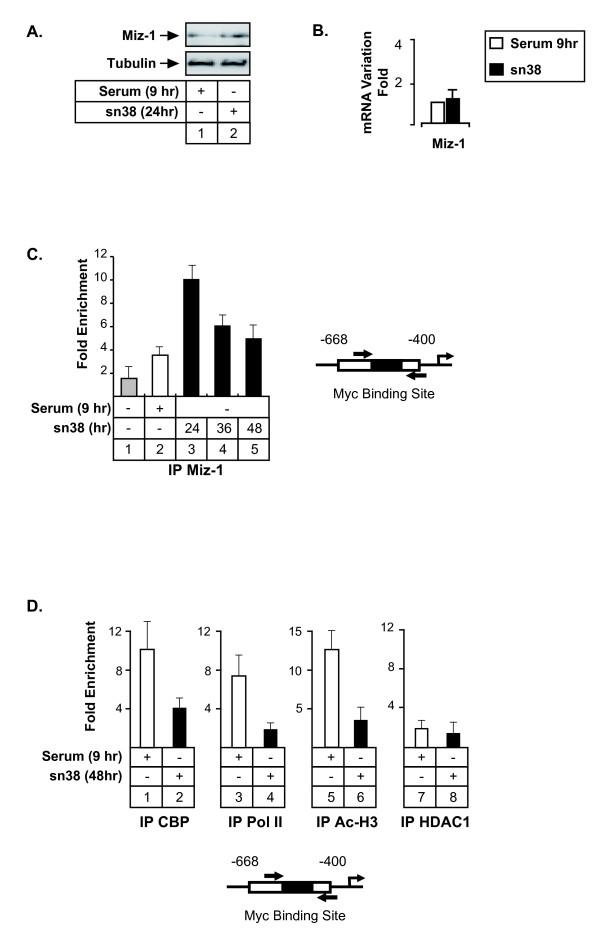
**Topoisomerase I inhibition induces the association of Miz-1 with the Aurora-A promoter and prevents the binding of transcriptional coactivators**. A-B. HCT116 were treated as described above and Miz-1 expression was analyzed by western blot (n = 3) or quantitative RT-PCR (B., n = 3 +/- sd). C. ChIP analysis of Miz-1 binding to the Aurora-A promoter in growing cells (lane 1), cells synchronized in G2 (lane 2) or following sn38 treatment. D. The recruitment on the Aurora-A promoter of CBP, RNA Pol II, HDAC1 and the acetylation of histone H3 were analyzed by ChIP using soluble chromatin prepared from cells synchronized in the G2 phase of the cell cycle (serum 9hr, lanes 1, 3, 5 and 7) or treated with sn38 (lanes 2, 4, 6 and 8, n = 3 +/- sd).

The Miz-1 transcription factor is a POZ-domain-containing zinc-finger protein that can form a transcriptional repressor complex with Myc to inhibit gene transcription [24]. In addition, it has also been proposed that Miz-1 functions as a transcriptional repressor in a Myc-independent manner through its association with cofactors such as BCL6 or Gfi-1 [25,26]. To determine if this protein was involved in Aurora-A inhibition, its expression was evaluated in HCT116 cells treated or not with sn38 (Figure 5A and 5B). Under these conditions, a weak increase in Miz-1 protein level was observed whereas no significant effect was detected on its mRNA expression. ChIP experiments performed in the G2 phase of the cell cycle showed that Miz-1 was associated with the -668/-400 region (Figure 5C, lane 2). By contrast, this protein was not significantly recruited to this gene in growing cells. Interestingly, Miz-1 recruitment was significantly increased following sn38 treatment (Figure 5C, lanes 3-5 and data not shown). Importantly, this binding was associated with a decreased recruitment of the CBP transcriptional coactivator, of the RNA type II polymerase and with a downregulation of histone H3 acetylation (Figure 5D, lanes 2, 4 and 6). We did not observe any recruitment of the HDAC1 histone deacetylase to this promoter.

Altogether, we concluded from these results that topoisomerase I inhibition induces a recruitment of Miz-1 to the Aurora-A promoter and decreases the binding of transcriptional coactivators.

### The Aurora-A promoter is located within SAHF foci following topoisomerase I inhibition

**Figure 6:**
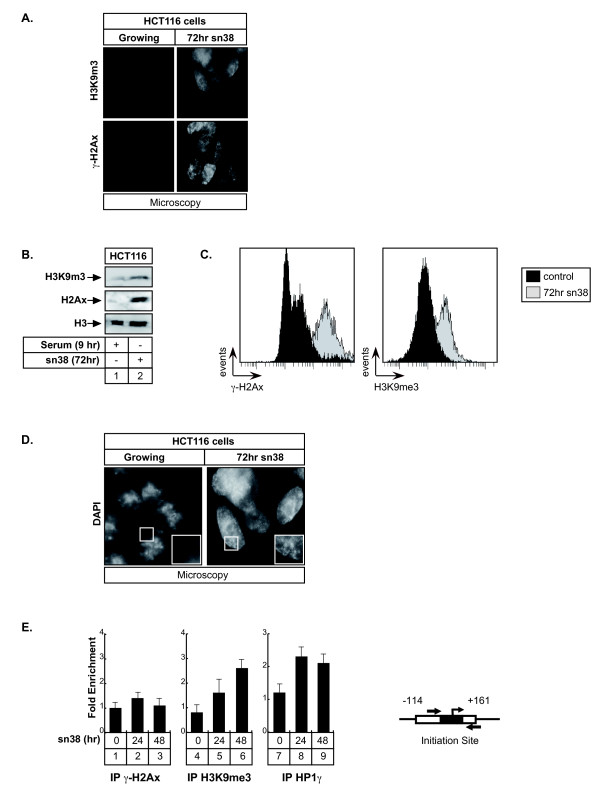
**The Aurora-A promoter is located within SAHF foci following sn38 treatment**. A, B and C. HCT116 cells were treated with sn38 as described above and H2Ax phosphorylation and H3K9 trimethylation were analyzed by immunofluorescence (A), western blot (B) or FACS (C, n = 3). D. SAHF formation was analyzed in growing cells or following treatment by immunofluorescence using DAPI staining (one experiment representative of three). E. HCT116 were treated as described above and the recruitment on the Aurora-A promoter of HP1γ, the trimethylation of histone H3K9 and the phosphorylation of histone H2Ax were analyzed by ChIP using soluble chromatin prepared from cells treated or with sn38 (n = 3 +/- sd)

We have recently shown that sn38 treatment induced senescence in colorectal cell lines (see [19,27] and text below). Senescence is an irreversible proliferation-arrest that is characterized by the formation of isolated heterochromatin foci called Senescence Associated Heterochromatin Foci (SAHF, [28]). SAHF foci contain marks of transcriptional silencing such as heterochromatin protein 1 (HP1) and tri-methylation of the lysine 9 of histone H3 (H3K9Me3). During senescence, proliferative genes such as E2F targets are compacted within these heterochromatin foci to prevent cell cycle progression, generally as a consequence of Rb-mediated silencing. To extend our results, we then determined if the Aurora-A promoter was included within these SAHFs foci. As a first approach, we used immunofluorescence and western blot experiments to shown that sn38 induced a global increase in H3K9 trimethylation in HCT116 cells. As expected, a significant phosphorylation of histone H2Ax was also detected, reflecting the induction of DNA double strand breaks following topoisomerase I inhibition (Figure 6A and 6B). Results were quantified by Facs analysis to show a significant increase of the two signals (Figure 6C). DAPI staining also showed an increase in the presence of punctuate heterochromatin foci in the nucleus of sn38-treated cells which were not detected in control conditions (Figure 6D). ChIP experiments were then used to determine if proteins involved in transcriptional silencing could be found associated with the proxymal promoter of the Aurora-A gene following treatment. Interestingly, results presented Figure 6E, lanes 4-9, showed that HP1γ was recruited to this gene in sn38-treated cells. In addition, we also noticed a significant increase in the amount of tri-methylated H3K9 on the proxymal Aurora-A promoter. By contrast, when ChIP experiments were repeated with an antibody directed against the phosphorylated form of histone H2AX, no signs of DNA double strand breaks were detected within this gene.

In light of these results, we concluded that the Aurora-A proxymal promoter is located within SAHF foci following genotoxic treatment and that its inhibition is probably related to the recruitment of cofactors involved in transcriptional silencing such as HP1γ and to the tri-methylation of H3K9.

### Topoisomerase I inhibition prevents centrosome separation

**Figure 7:**
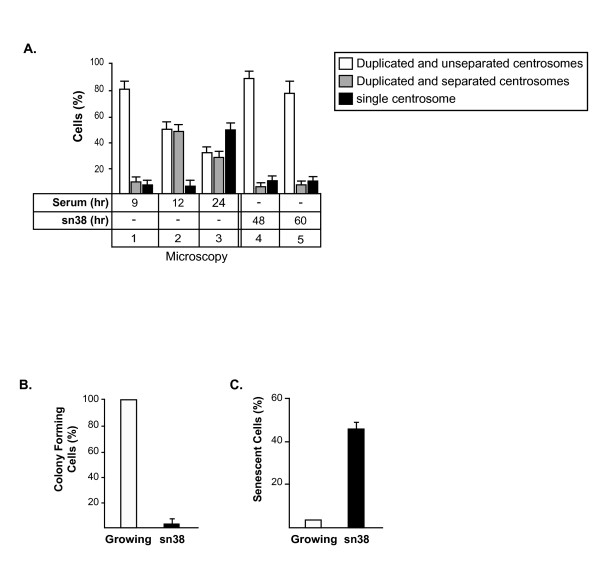
**Topoisomerase I inhibition prevents centrosome separation**. A. HCT116 cells synchronized or treated with sn38 as described above were subjected to immunofluorescence analysis. Centrosomes were detected by staining with monoclonal antibody to γ-tubulin. ~ 100 cells were analyzed for each experiment, (n = 3 +/- sd). B. HCT116 cells were treated with sn38 (5 ng/ml, 12.5 nM) and further grown for 7-9 days. Colony formation was then counted using an inverted microscope, and for each cell line, growth of non treated cells was set up at 100%. Clonogenic survival was then plotted as a fraction relative to these untreated cells (n = 5 +/- sd). C. In parallel, the percentage of senescent cells was evaluated as the number of cells expressing SA-β-gal activity (n = 3).

It has been shown that Aurora-A is involved in the maturation and separation of centrosome during progression from S phase towards mitosis [29]. To determine if topoisomerase I inhibition prevents this maturation, centrosome formation was analyzed by immunofluorescence and γ-tubulin staining. When cells were synchronized in the G2/M phase of the cell cycle, the centrosomes were effectively stained as a doublet and Aurora-A was essentially localized on the centrosomes. As expected, when cells were treated with sn38, Aurora-A became undetectable by immunofluorescence (data not shown). Interestingly, genotoxic treatment dit not prevent centrosome duplication, however, no separation was observed under these conditions (Figure 7A). Probably as a consequence of the absence of centrosomal separation and of progression towards mitosis, we observed using clonogenic assays that sn38 induced a complete inhibition of cell proliferation (Figure 7B). Using beta-galactosidase staining, we also noticed an induction of senescence following genotoxic treatment (Figure 7C).

Thus, we concluded from these results that topoisomerase I inhibition prevents centrosome separation, probably as a consequence of Aurora-A inhibition, and that this leads to G2 arrest and senescence induction.

## Discussion

In this study, we have shown that the Aurora-A gene is inhibited upon topoisomerase I inhibition. In normal conditions, the Myc transcription factor is recruited to the promoter of the Aurora-A gene in association with its binding partner Max. Following topoisomerase I inhibition, Mad proteins increase, the association of Myc and Max with the Aurora-A promoter is inhibited, the Mad and Miz-1 proteins are recruited to DNA and this is followed by transcriptional downregulation. Probably as a consequence of the downregulation of the Myc-Aurora A pathway, genotoxic treatment also prevented centrosome separation. In light of these results, we propose that the downregulation of the Aurora-A gene is one of the essential events of G2 arrest occurring in response to topoisomerase I inhibition.

Gene transcription is regulated at multiple steps including DNA binding of transcription factors, recruitment of the basal transcriptional apparatus and elongation of mRNA synthesis. Activation is also affected by several complexes that affect nucleosomal structure [30] such as histone acetyltransferase (HATs) proteins and chromatin remodeling complexes. In light of our results, we speculate that Myc is associated with Max on the Aurora-A promoter to allow the recruitment of transcriptional coactivators previously shown to be associated with Myc, such as TRAPP, a subunit of the TIP60 histone acetylase complex, or TIP48 and TIP49, two ATPases involved in chromatin remodeling [31]. In addition, Myc can also regulate the elongation program through its association with the P-TEFb complex and cdk9 [32,33]. It will be interesting to determine if Myc regulates the elongation process on the Aurora-A gene as previously reported on the cad promoter [32,33], or if its effects rely on the recruitment of histone acetylases and chromatin remodeling complexes. We have previously shown that topoisomerase I inhibition induced senescence in colorectal cancers [19]. It has been proposed that this program is associated with chromatin reorganization of proliferation genes into senescence-associated heterochromatin foci (SAHFs) [28]. Silencing depends on the retinoblastoma pathway and is associated with enhanced histone H3 tri-methylation and recruitment of the HP1 protein on proliferative genes. Interestingly, we have effectively observed that SAHFs are present in colorectal cancer cells treated with sn38 and that topoisomerase I inhibition is associated with the recruitment of HP1γ and trimethylation of H3K9me3 on the Aurora-A promoter. Since Miz-1 interacts with transcriptional repressors such as Gfi-1, Dnmt3a or BCL6 to downregulate gene transcription [25,26,34,35], a Miz repressor complex could inactivate the Aurora-A promoter by initiating SAHFs formation on this gene. Since SAHFs formation has been initially described to be associated with transcriptional silencing induced by the Rb protein, our results also suggest that this gene might be a target of this suppressor pathway. Note however that we have not been able to detect the expression of the p16INK4 protein in our conditions. Thus, if the Aurora-A promoter is regulated by the Rb protein following sn38 treatment, this does probably not rely on p16INK4.

**Figure 8:**
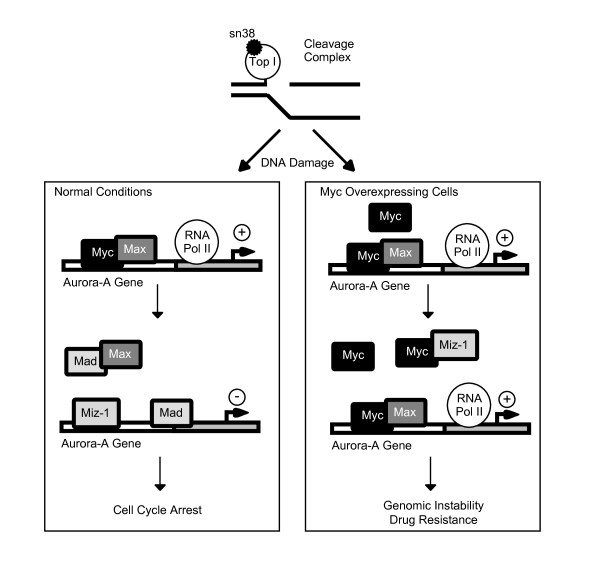
**Proposed hypothesis for the role of the Myc-Aurora-A pathway in response to topoisomerase I inhibition**. In normal conditions, the Aurora-A gene is activated and Myc binds to its promoter in association with Max. Upon treatment, sn38 binds to the topoisomerase I and induces the formation of cleavage complexes. This induces a dowregulation of Myc and an increase in the expression of Mad. Myc/Max binding is inhibited and Mad and/or Miz-1 binds to the Aurora-A promoter. Although this remains to be shown, we speculate that these proteins associates with transcriptional inhibitors such as Gfi-1 or Dnmt3a to induce SAHF foci and Aurora-A downregulation. In colorectal tumors overexpressing Myc, the Myc/Max complex remains associated with the Aurora-A promoter due to a high level of expression and to a downregulation of Mad and Miz-1 expression. As a consequence, Aurora is overexpressed, this protein is not inhibited by topoisomerase I inhibitors and this induces drug resistance.

Accumulating evidences indicate that Myc or Aurora A overexpression is associated with chromosomal instability [10,12,36]. Since both oncogenes play an important role in colorectal cancers, we have started to determine if this oncogenic pathway is associated with genomic instability in colorectal cancers. Preliminary data indicate that the vast majority of colorectal tumors showed a high degree of aneuploidy correlated with an enhanced expression level of Myc and Aurora A. A downregulation of Miz-1 and of the p21waf1 cell cycle inhibitor was also observed. In light of these results, we propose the hypothesis that the dysregulation the Myc-Aurora A pathway is an important event leading to genomic instability through the bypass of the G2/M checkpoints. We speculate that tumors expressing abnormal levels of Myc together with a high expression of Aurora-A might be resistant to DNA-topoisomerase I inhibitors such as irinotecan. The downregulation of the p21waf1 protein is also probably an essential event to allow the inactivation of the senescence program. For this reason, we propose the hypothesis that the coordinated overexpression of Myc and Aurora-A, together with a downregulation of Miz-1 should be tested as a prognosis signature of poor responses to topoisomerase I inhibitors (Figure 8). This signature should help to define in advance the subsets of tumors that will fail to respond to chemotherapy.

## Conclusions

Following DNA damage, the ATM/ATR/chk pathway is activated to induce the upregulation of the p53 tumor suppressor and the consequent activation of the p21waf1 gene. In parallel, the cdc25 phosphatases are inactivated, leading to cdk inhibition and cell cycle arrest. Using colorectal cancer cell lines, we show that the Aurora-A gene is also downregulated following topoisomerase I inhibition and that this effect is probably related to a decreased recruitment of the Myc transcription factor to its promoter. We propose the hypothesis that tumors expressing high levels of the Myc-Aurora-A pathway might be resistant to topoisomerase I inhibitors.

## Competing interests

The authors declare that they have no competing interests.

## Authors' contributions

JC, SC performed most experiments, including mRNA, protein, ChIPs and cell cycle analysis and SC also helped editing the manuscript. AV made senescence and proliferation assays whereas MBT did cloning experiments. SG and IV analyzed the data. EG, CP, BB and OC provided the suggests and wrote the paper. All authors read and approved the final manuscript.
